# High Resolution Melt Analysis (HRMA); a Viable Alternative to Agarose Gel Electrophoresis for Mouse Genotyping

**DOI:** 10.1371/journal.pone.0045252

**Published:** 2012-09-19

**Authors:** Nicole Thomsen, Radiya G. Ali, Jehangir N. Ahmed, Ruth M. Arkell

**Affiliations:** Early Mammalian Development Laboratory, Research School of Biology, The Australian National University, Canberra, Australian Capital Territory, Australia; Wellcome Trust Centre for Stem Cell Research, United Kingdom

## Abstract

Most mouse genetics laboratories maintain mouse strains that require genotyping in order to identify the genetically modified animals. The plethora of mutagenesis strategies and publicly available mouse alleles means that any one laboratory may maintain alleles with random or targeted insertions of orthologous or unrelated sequences as well as random or targeted deletions and point mutants. Many experiments require that different strains be cross bred conferring the need to genotype progeny at more than one locus. In contrast to the range of new technologies for mouse mutagenesis, genotyping methods have remained relatively static with alleles typically discriminated by agarose gel electrophoresis of PCR products. This requires a large amount of researcher time. Additionally it is susceptible to contamination of future genotyping experiments because it requires that tubes containing PCR products be opened for analysis. Progress has been made with the genotyping of mouse point mutants because a range of new high-throughput techniques have been developed for the detection of Single Nucleotide Polymorphisms. Some of these techniques are suitable for genotyping point mutants but do not detect insertion or deletion alleles. Ideally, mouse genetics laboratories would use a single, high-throughput platform that enables closed-tube analysis to genotype the entire range of possible insertion and deletion alleles and point mutants. Here we show that High Resolution Melt Analysis meets these criteria, it is suitable for closed-tube genotyping of all allele types and current genotyping assays can be converted to this technology with little or no effort.

## Introduction

Worldwide efforts to create targeted, gene-trap and chemically induced alleles of each mouse gene and to generate GFP reporter and *Cre* transgenes from a multitude of tissue specific promoters means that many laboratories now maintain a plethora of genetically altered strains of mice [1], [2]. Many of these strains need to be propagated via heterozygous carriers (for example due to the embryonic lethality associated with the homozygous locus, or to minimise the chance of a randomly integrated transgene causing a deleterious effect) and consequently all progeny need to be genotyped to identify carrier animals. Additionally, many of the transgenic strains are useful only when crossed with a second genetically altered strain (for example cross between a *Cre*-transgene and a conditional targeted mutation), again meaning that progeny need to be genotyped (at more than one locus) to identify compound heterozygous animals. The combined effect is that the genotyping of mouse strains occupies a significant component of research effort in many laboratories.

Despite the many advances in mouse genetics and genomics that have enabled the expanded production and use of genetically unique mouse strains, the techniques used for genotyping have remained relatively static. The method most commonly employed to genotype mice remains PCR amplification of allele-specific sequences from mouse genomic DNA followed by separation of the products by gel electrophoresis [3]. Both the isolation of genomic DNA and PCR set-up have been streamlined in recent years, for example by the use of tissue lysis buffers compatible with PCR (without the need for precipitation of the genomic DNA) [4] and by the generation of PCR systems in which the reaction buffer, dNTP’s, Taq polymerase and inert gel loading dyes are all incorporated into a commercially prepared, one-tube mixture [5]. Combined with the use of multi-channel pipettes, multi-well plates, effective sealing methods and heated-lid PCR machines the genomic isolation and PCR phase of the genotyping procedure can be achieved with relatively little researcher ‘hands-on’ time.

Some time-saving modifications of the electrophoresis component of genotyping assays have also been introduced. Pre-poured agarose gels, multi-channel pipette compatible gels, buffer free electrophoresis and automated electrophoresis can all increase through-put [6]. Nonetheless, for many laboratories, the time required to pour high quality agarose gels, to load samples, to obtain a photographic record of the gel analysis and score sample results remains lengthy. Recently many new techniques that utilize automated post-PCR analysis to discriminate alleles have been developed. The majority of these techniques however discriminate only single nucleotide polymorphisms (SNPs) and not the amplicon size differences that are generally separated by agarose electrophoresis when genotyping genetically modified mouse strains [7], [8], [9]. One method that is able to discriminate both SNPs and fragment size differences is DNA melting analysis; raising the possibility that the need for post-PCR electrophoresis could be eliminated altogether.

The kinetics by which double stranded DNA (dsDNA) separates into two single strands (ssDNA) upon heating (known as denaturation or DNA melting) is affected by base composition [10] and has long been used to study DNA characteristics. For example this principle forms the basis of identifying DNA sequence and sequence-length polymorphisms in techniques such as Single Strand Conformational Polymorphism (SSCP) analysis [11]. During the development of real-time PCR the ability to analyse the DNA melting kinetics of amplicons into which a fluorescent dye was intercalated became incorporated into real-time machines as a means of optimising reactions [10], [12]. This feature of a real-time PCR machine and incorporation of the fluorescent intercalating dye, SYBR green, during PCR has previously been used to replace SSCP discrimination of Simple Sequence Length Polymorphisms (SSLP) for mouse genotyping [13], [14]. SYBR green fluoresces when intercalated into double-stranded DNA (dsDNA) but not single-stranded DNA (ssDNA) generating differential fluorescence emission dependent on association with double-stranded or single-stranded DNA [15], [16]. Thus, when dsDNA incorporated with SYBR green is heated, the dye is released from the DNA duplex resulting in a decrease in fluorescence which allows DNA melting to be visualised. Different DNA duplexes can be distinguished as each will give a characteristic melting profile. More recently, technical advances (such as the development of fluorescent saturating dyes and of machines that precisely regulate temperature and carry out rapid repeated measurements) have enabled the development of the rudimentary melt curve analysis into the technique now known as High Resolution Melt Analysis (HRMA) [17].

This technique is rapidly gaining in popularity and many publications report its use for the detection of Single Nucleotide Polymorphisms (SNPs) or other mutation types in a range of fields [18], [19], [20] and to discriminate RT-PCR products [21]. The technique should theoretically also be suitable for the genotyping of mouse strains that carry a targeted or gene-trap mutation, a transgene insertion or other sequence length based polymorphism. The analysis of PCR products by HRM (rather than gel electrophoresis) would confer several advantages. These include practical issues such as time saving (since the time required to pour, load, record and analyse gels is avoided) and the fact that data analysis can be performed automatically decreasing the chance of errors incurred when transferring data from a gel to table format. Furthermore, HRM analysis takes place in the same tube as the PCR without the need to open the tube between amplification and product analysis. These so called closed-tube methods confer the advantage of significantly decreasing the risk of future PCR contamination. Closed-tube methods also lend themselves to high-throughput applications and since melting analysis can be accomplished in under ten minutes per plate of samples, HRMA can be high-throughput. Finally, HRMA has the advantage that it is non-destructive and so, if HRMA fails or is ambiguous, trouble-shooting can be carried out by gel analysis or sequencing of the reaction products.

Although the discrimination of Fragment Length Polymorphisms and Sequence Variants by HRMA appears to offer many advantages over gel electrophoresis, the perceived need to re-optimise genotyping assays for a new technique is a significant impediment to the development of new laboratory protocols. In the simplest scenario, pre-existing genotyping assays could be converted for HRMA by the addition of the fluorescent dye post-PCR directly before melting. This protocol however removes the closed-tube advantage and is not the preferred approach. Here we examined whether PCR products already used to genotype mice are readily amenable to discrimination by HRMA. We attempted to convert 25 genotyping assays currently in use in our laboratory. We found that in each case no alteration to existing PCR conditions was required and that the necessary allele classes could readily be discriminated. HRMA appears to be a viable alternative to gel electrophoresis analysis of PCR amplicons for the genotyping of mouse strains.

## Materials and Methods

### Ethics Statement

Mice were maintained according to Australian Standards for Animal Care under protocol A2011/63 approved by The Australian National University Animal Ethics and Experimentation Committee for this study.

### Samples and DNA extraction

Ear biopsy tissue was collected into 50 µL of lysis solution (TE, pH 7.5 and 0.1% Tween-20) containing 2 µg/µL proteinase K and DNA isolated as previously described [13]. PCR was performed from 1/20^th^ of the diluted sample.

### Assay Design and PCR

All assays were designed to generate two differential products. For those assays in which suitable primers were not already available, primers were designed such that all primers common to an assay had a melting temperature (Tm) within 2°C of one another. These conditions were generally met by designing a 17 mer with (8 G/C and 9 A/T) or (9 G/C and 8 A/T) nucleotides. To decrease the probability of non-specific priming a minimum of three out of the five 3′ most nucleotides were a G or a C. The primers specific to each assay are listed in Table S1. Products to be analysed by electrophoresis were generally obtained by amplification from genomic DNA (30 ng) with 0.025 units/µL ThermoPrime Plus DNA Polymerase (Thermo Scientific) in a total volume of 15 µL in the presence of 0.8 µM each oligonucleotide and 20 µM each di-Nucleotide Triphosphate (dNTP). The PCR buffer (AbGene ReddyMix™, Thermo Scientific; Cat. No. AB-0575LDDCA) led to final reaction conditions of 75 mM Tris-HCL (pH 8.8 at 25°C), 20 mM (NH_4_)_2_SO_4_, 0.01% (v/v) Tween 20 and 1.5 mM MgCl_2_ and also contained an inert gel loading dye. PCR was performed in 96 Well Clear, Flat Top PCR plates (Axygen; Cat. No. PCR-96-FLT-C) and the plate was sealed with an Easy Pierce Heat Sealing Film (Axygen; Cat. No. MF-111). Products to be analysed by HRMA were generally obtained by amplification from genomic DNA (30 ng) with 0.025 units/µL IMMOLASE™ DNA Polymerase (Bioline) in a total volume of 10 µL in the presence of 0.8 µM each oligonucleotide and 20 µM each di-Nucleotide Triphosphate (dNTP) and a 1∶10 dilution of LCGreen® Plus dye (Idaho Technologies Inc.). The PCR buffer (ImmoMix™, Bioline) led to final reaction conditions of 67 mM Tris-HCL (pH 8.3 at 25°C), 16 mM (NH_4_)_2_SO_4_, 0.01% (v/v) Tween 20 and 1.5 mM MgCl_2_. To avoid evaporation during the HRM process each reaction was covered by a drop (5–10 µL) of mineral oil prior to PCR. PCR was performed in Hard-Shell® 96 well PCR Plates (BioRad; Cat. No. HSP-9665) covered with MicroAmp Optical Adhesive Film (ABI). All PCRs were performed in an Eppendorf Mastercycler using the following Touchdown PCR program: the initial annealing temperature of 65°C (TD65) or 60°C (TD60) was decreased by 0.5°C per cycle for 19 cycles and held at 55°C for 30 seconds for the subsequent 19 cycles, for all cycles denaturation was performed at 94°C for 30 seconds and extension was carried out at 72°C for 30 seconds.

### Analysis of PCR Products

PCR products were analyzed by horizontal electrophoresis through 2% or 3% agarose (SeaKem LE, Cambrex) in 1× TBE (0.1 M Tris-HCL, 0.09 M boric acid and 0.001 M EDTA) gels containing a 1∶20,000 dilution of Red Safe nucleic acid staining solution (InTron Biotechnology). The gels were electrophoresed at 6 V/cm for 20 minutes in 1× TBE and reaction products viewed and recorded in a Versa Gel Doc System (BioRad). For High Resolution Melt Analysis the 96 well plate containing PCR products was placed directly into a LightScanner® (Idaho Technologies Inc.) and samples melted from 60 to 95°C at a rate of 0.1°C/sec. The data were analysed with LightScanner software (Idaho Technologies Inc.).

## Results

### Genotyping Assays Suitable for Conversion to HRMA

**Figure 1:**
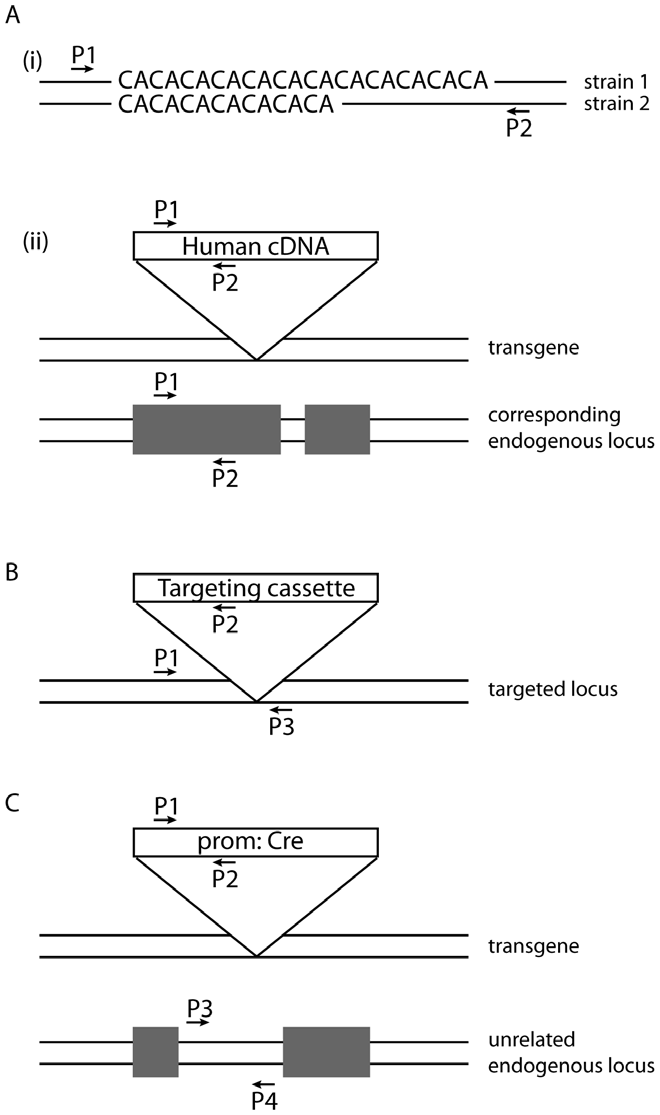
Plus/plus genotyping assay design. (A) Two-primer assays are suitable when common sequences flank a divergent sequence. The divergence may for example be due to (i) strain specific polymorphisms (such as microsatellite markers), or (ii) genomic incorporation of an orthologous DNA sequence and may generate products of differing size and/or sequence. (B) Three-primer assays are appropriate when the site of incorporation of exogenous DNA is known. The P1/P2 primers will specifically amplify a product from the modified locus by virtue of the exogenous DNA specific P2. The P1/P3 primers will only amplify a product from the wild type locus because PCR conditions are set to favour the short product and inhibit the amplification of the theoretically possible but much larger product from the altered locus. (C) Four-primer assays are useful when the location of the exogenous DNA is unknown and the exogenous sequence is unrelated to the mouse genome. The P1/P2 primers are unique to the exogenous DNA and P3/P4 can correspond to any unrelated region of the mouse genome. P: primer.

Genotyping of mouse strains can theoretically use an assay which produces only one amplicon from the genetically altered animal (for example a transgene specific amplicon). HRMA could identify carrier animals on the basis of the presence or absence of a product in the HRM read out. Such plus-minus assays are not however generally recommended for mouse genotyping due to the high false negative rate that can occur for example due to poor DNA quality. To avoid this problem all of the assays to be tested by HRMA were designed according to the principles shown in Figure 1 so that each assay generates two differential allele products. As shown in Figure 1, the genotyping assays examined here can be divided into three categories based on the number of primers included in the PCR component of the assay. (1) Two-primer assays: only two primers are required to generate differential products from each allele when genotyping Short Sequence Length Polymorphisms (SSLPs). These may be endogenous (in the case of amplification across microsatellites polymorphic between the parental strains; Figure 1Ai) or may arise due to the incorporation of a transgene derived from a different species (for example a transgene consisting of Human genomic DNA) where intron length varies between the exogenous and endogenous locus. Alternatively products with differential melt profiles could arise when products of the same length have a different sequence composition because of differences between the mouse and the transgene exon sequence (Figure 1Aii). (2) Three-primer assays can be used to genotype mice with a targeted mutation, a gene-trap mutation, or transgene insertion in which the integration site of exogenous DNA is known (Figure 1B). (3) Four-primer assays can be used when a transgene carrying sequences without a mouse orthologue (such as a *Cre*, *LacZ* or *GFP* gene) is incorporated and in which the integration site of exogenous DNA is unknown. In this case two primers amplify a transgene specific product and two primers amplify an unrelated product which serves as an internal positive control (Figure 1C).

### HRMA can Distinguish Both Homozygous Products from the Heterozygous Product

**Figure 2:**
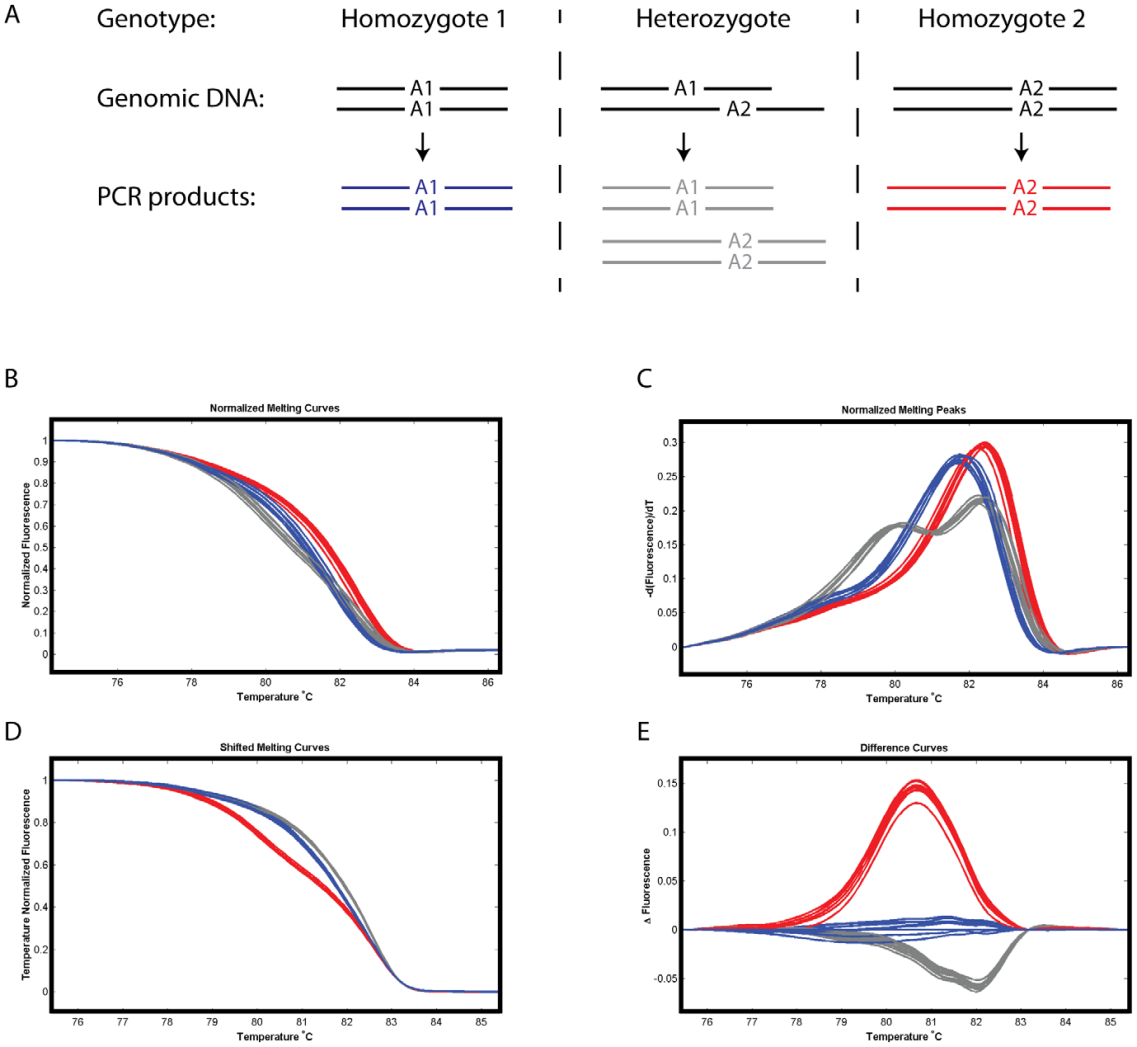
Heterozygous animals have a melt profile distinct from that of either homozygote. (A) Diagram representing the amplicons derived from genomic DNA that is homozygous for one of two differently sized alleles (Allele 1 or Allele 2) or heterozygous for the two alleles. The heterozygote PCR product will predominantly contain homoduplexes of either Allele 1 or Allele 2 because heteroduplex formation is less favourable due to length and/or sequence differences between the allele amplicons. (B–E) HRM data following a two primer PCR to amplify the SSLP marker microsatellite D8Mit155 from mice derived from crosses between the BALB/c and FVB inbred strains such that progeny may be homozygous for one of the parental alleles or heterozygous for the two alleles. D8Mit155 spans a TG dinucleotide repeat and produces an amplicon of 139 bp from the BALB/c genome and 151 bp from the FVB genome. The melting peak analysis readily distinguishes the heterozygote animals which exhibit two melting point peaks. Blue: PCR products derived from an animal homozygous for Allele 1, Grey: PCR products derived from an animal heterozygous for Allele 1 and 2, Red: PCR products derived from an animal homozygous for Allele 2.

Mouse breeding protocols may require that a heterozygous carrier be distinguished from both of the possible two homozygous allele types, for example, a cross between two animals each of which carries one copy of the altered allele (a heterozygous carrier) will produce 25% of progeny homozygous for the wild-type allele, 50% heterozygous for the wild-type and altered allele and 25% of progeny homozygous for the altered allele. A genotyping PCR may therefore result in three classes of products, two of which consist only of one allelic product (i.e. Allele 1 or Allele 2 homoduplexes) and one that contains a mixture of Allele 1 and Allele 2 homoduplexes. When conventional genotyping by electrophoretic analysis of PCR products is used, the heterozygote samples are readily identified by the presence of both of the differently sized Allele 1 and Allele 2 products. As shown in Figure 2, HRMA allows the distinction of heterozygotes (from either of the homozygotes) since the melting profile of this sample is distinct from that of either homozygote. This occurs because each of the homoduplexes has differing stabilities and the melting curve of the heterozygote sample is a combination of the melting profile of each homoduplex contained within the sample.

**Table 1:** Assay conversion.

Assay	Amplicon Sizes (bp)	No Optimisation	Δ Primer Ratio	Preferred Analysis Method
**Two-Primer Assays**				
D6Mit268	110, 123	+++	NT	NMP
D6Mit235	180, 196	+++	NT	NMP
D6Mit213	116, 150	+++	NT	NMP
D6Mit201	106, 148	+++	NT	NMP
D6Mit83	130, 150	+++	NT	NMP
D6Mit104	146, 158	+++	NT	FDC
D7Mit76	202, 224	+++	NT	FDC
D7Mit247	100, 130	+++	NT	NMP
D7Mit276	114, 132	+	+++	NMP
D7Mit69	232, 238	+	+++	FDC
D8Mit155	139, 151	+++	NT	NMP
D9Mit89	148, 160	+++	NT	NMP
D9Mit12	82, 90	+++	NT	FDC
D9Mit214	116, 138	+++	NT	FDC
FIC	437, 536	+++	NT	NMP
FIT	400, 453	+++	NT	NMP
Ube1 sex assay	132, 138	+++	NT	NMP
**Three-Primer Assays**				
FIN	278, 400	+++	NT	NMP
RRF	279, 383	+++	NT	NMP
RRFU	279, 334	+++	NT	NMP
NLZ	280, 700	−	−	FDC
ND6	240, 470	+++	NT	FDC
Z5N	107, 130	+++	NT	NMP
**Four-Primer Assays**				
Mnet	249, 450	+++	NT	NMP
K5C	249, 520	+++	NT	NMP
K5S	249, 400	+++	NT	NMP

Amplicon sizes are approximate. Assays were performed in the HRMA reaction conditions and examined by gel electrophoresis and scored as follows: +++; both amplicons produced in equivalent amounts, +; both amplicons produced but one is favoured, −; one or both of the amplicons is not produced. Assays that did not score +++ were optimized with altered primer ratios and again assessed by electrophoresis. NT: not tested, NMP: Normalized melting peak, FDC: Fluorescence difference curve.

The software associated with commercially available HRMA systems offers different methods of viewing the data which assist with distinguishing genotypes even when the melting profile of each sample is similar. The normalized melting curve raw data are represented as the amount of fluorescence at each given temperature (called the normalized melting curve, Figure 2B). The normalized data can be transformed in various ways to increase the ease of discriminating genotypes. By calculating the negative first derivative of each measurement (−dF/dT) the plot reveals melting temperature maxima (called the normalized melting peak, Figure 2C). When the Allele 1 and Allele 2 homoduplexes have different melting peaks heterozygote samples are readily identified by the presence of two peaks in this plot. A second data transformation adjusts each of the normalized melting curves so that all curves meet the background fluorescence at the same temperature (called temperature shifted melting curve, Figure 2D). A third analysis method involves plotting the fluorescence difference between normalized melting curves. Here one genotype is chosen as a reference (user defined and usually one of the homozygote genotypes is chosen) and the difference between each curve and the reference is plotted against temperature (called the fluorescence difference curve, Figure 2E). The reference curve becomes a horizontal line at zero and the other genotypes cluster along different paths for easy visual discrimination of the genotype classes. The use of these analysis methods mean that it is often possible to discriminate the required genotype classes even when PCR efficiencies vary between amplicons and/or samples. In mouse genotyping the samples will generally require distinguishing heterozygous animals from at least one of the homozygous alleles (often homozygous wild-type) and the melting peak analysis is therefore generally useful because of the presence of two peaks in heterozygous samples. In 19 of the 26 assays examined here we found that melting peak analysis discriminated the required allele. For the remaining 7 assays the results were easier to visualise when the sample producing the intermediate melt curve was set as the reference and fluorescence difference curves were generated (Table 1).

**Figure 3:**
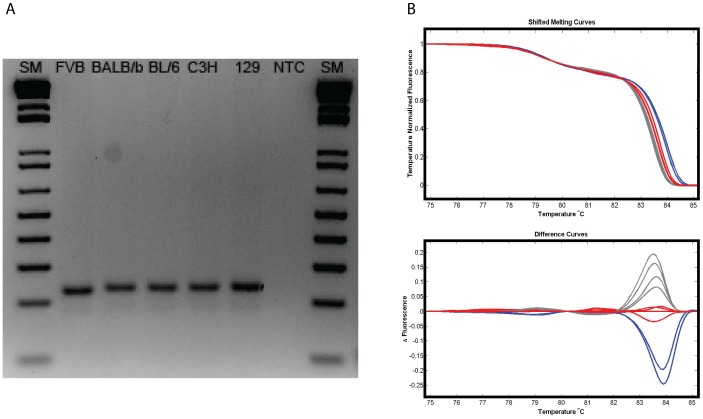
Small changes in fragment length or sequence are sensitively detected by HRMA. (A) Agarose gel electrophoretic analysis of products derived from amplification of SSLP marker microsatellite D7Mit69 from 5 inbred strains of mice. The predicted allele sizes are: FVB; 230 bp, BL/6 and BALB/b; 236 bp, C3H and 129; 238 bp. (B) High Resolution Melt Analysis of the same products showing that the three allele sizes are readily discriminated using either Shifted melting curve or Difference curve analysis. Blue: FVB, Red: C3H and 129, Grey: BALB/b and BL/6. SM: 1 Kb+ DNA ladder, NTC: No template control.

### Assay Conversion

To determine the ease with which genotyping assays already in use within the laboratory can be converted for HRMA analysis we first tested each of the 26 assays studied here with HRMA specific reagents, but made no other changes to our protocol. Each of the assays for conversion had previously been optimized in an (NH_4_)_2_SO_4_ buffer that was supplied as a pre-mix that contained the other reaction components as well as an inert agarose gel loading dye (ReddyMix™). For HRMA analysis this reagent was replaced with one that contained similar reaction components but which lacked the inert gel loading dye (ImmoMix™). The saturating fluorescent dye, LCGreen® Plus, was added to the reaction prior to commencing PCR and reactions were set up using plasticware compatible with fluorescent measurements (optical 96 well plates and optical seals). All other reaction components and conditions (including primer concentrations and thermo-cycling programs) were held constant. The resulting amplicons were examined by agarose gel electrophoresis to check that products were amplified in this reagent mix as expected. As shown in Table 1, 17 two-primer assays, 6 three-primer assays and 3 four-pimer assays were tested in this manner and the majority of assays (23/26∶88%) resulted in the two expected amplicons. A further two assays showed bias against a specific allele and this was rectified by altering primer ratios, leaving one assay which did not work in the ImmoMix™ buffer system. The 25 assays that gave two discernible amplicons were analysed by HRM and in all cases the amplicons could be distinguished by HRMA (Table 1). For the remaining assay, we used the original buffer system with the inert gel loading dye for PCR and added LC Green after PCR and found that HRMA was able to discriminate the products.

Many reports that describe HRMA for SNP typing emphasize the need to limit amplicon size in order to maximize the effect of the SNP on the melt profile and recommend amplicons of ∼50 bp. In contrast to SNP genotyping, the fragment length polymorphism assays routinely used to genotype mouse strains often have comparatively disparate amplicons that may vary considerably in size (eg by 20–50 bp) and/or sequence composition. To determine whether amplicon size or the relative difference between amplicons within an assay influenced the success of HRMA discrimination, assays with a range of characteristics were selected for HRMA conversion. Table 1 shows that the assays tested here included amplicons that ranged in size from 82 bp to 700 bp and that the allele sizes within an assay differed by as little as 6 and as many as 420 bp in length. Figure 3 shows an assay in which the fragment length polymorphisms varied by 2, 6 and 8 bp in size and are at the limit of distinction by electrophoresis. These fragments are readily distinguishable by HRMA indicating that HRMA is at least as sensitive as gel electrophoresis. It therefore seems that providing existing assays generate differential allele products that can be discriminated by electrophoresis the assay should be amenable to analysis by High Resolution Melting.

## Discussion

Mouse genotyping is time consuming, expensive and if inaccurate can lead to costly errors in mouse maintenance. Currently the method most commonly employed to genotype mice is PCR amplification of specific sequences from mouse genomic DNA followed by separation of the products by gel electrophoresis. HRMA is a relatively new technique which is rapidly becoming adopted for SNP analysis. Here we show that HRMA is also a viable alternative to agarose electrophoresis for discriminating Fragment Length Polymorphisms and Sequence Variants of the sort that can be obtained from naturally occurring polymorphisms and from transgenic, targeted and gene trap mouse strains. In combination with the well documented ability of this technique to detect SNPs HRMA therefore represents a common platform amenable to the genotyping of all mouse strains.

In deciding to switch to a new technology most laboratories will consider not just whether the technique works but also (i) whether it represents a long term time and/or cost saving, and (ii) the effort required to establish the new technique. HRMA removes the researcher time required to pour, load, document and analyse agarose gels and therefore represents a time and labour-cost saving. In the study presented here we found that reagent costs were similar between gel electrophoresis and HRMA since, for example, the cost of fluorescent dyes and HRMA compatible plasticware was offset by the savings on agarose and reagents for gel documentation. Overall, because of the saving on labour, HRMA represents an ongoing time and cost saving.

The second consideration concerns the investment required to trial and establish the new technique. New techniques seem more likely to be widely adopted when researchers do not have to commit to purchasing expensive machines prior to commencing the technique. The types of machines that can perform HRMA have been described elsewhere [22] but include many Real-time PCR machines (equipped with suitable analysis software) of the type routinely available in most research departments. Many researchers therefore may avoid purchasing a dedicated HRMA machine until the technique is firmly established. In addition to this feature, the study described here shows that scant effort is required to adapt pre-existing genotyping assays for HRMA. Providing that an assay generates differential products these products should be distinguished by HRMA. Some optimisation may be required if different PCR buffer/polymerase reagents are used for electrophoresis and HRMA. If current PCR protocols do not use a buffer that incorporates an inert loading dye then it may be anticipated that the only alterations required are the inclusion of the fluorescent dye and mineral oil at PCR set up and the use of optical grade plastic ware. If current PCR reagents do include an inert gel loading dye then the simplest approach to adapt current protocols for HRMA is to identify a PCR buffer/polymerase system that is as similar to the current system as possible, but which omits the inert loading dye. Many companies supply the same PCR buffer/polymerase system with and without an incorporated gel loading dye providing the ideal comparative system. Although small (50 bp) amplicons are often recommended for SNP discrimination with HRMA the assays examined here involved fragments from 82 bp to 700 bp, suggesting there is no need to design small amplicon assays before embarking on HRMA. Additionally, the difference in size of the fragments within an assay varied greatly (from 6 bp to 420 bp) and did not appear to influence the success of HRMA.

In conclusion, HRMA offers several advantages over gel electrophoresis for mouse genotyping. The main advantages are that it represents a single platform on which all types of induced genome alterations can be genotyped and enables mouse genotyping in a closed-tube system. This high-throughput technique saves on labour and does not entail a commitment to the ongoing purchase of company-specific, dedicated consumables making it cheaper than current electrophoresis protocols. Combined with the demonstrated ease of adapting established mouse genotyping protocols for HRMA it has the potential to be widely adopted and to increase the efficiency of mouse genetics.

## Supporting Information

Table S1 **Primers and PCR conditions.** Primer sequences for microsatellite markers obtained from the Mouse Genome Informatics website.
(DOCX)
