# Supplementary material for: High Resolution Melt Analysis (HRMA); a Viable Alternative to Agarose Gel Electrophoresis for Mouse Genotyping
Source: PLoS One. 2012 Sep 19;7(9):e45252. doi: 10.1371/journal.pone.0045252 (PMC3446988; doi:10.1371/journal.pone.0045252)
Supplement: Table S1 — Primers and PCR conditions. Primer sequences for microsatellite markers obtained from the Mouse Genome Informatics website. (DOCX) [file pone.0045252.s001.docx]

| Assay | Left Primer/s (Tm°C) [Arkell oligo number] | Right Primer/s (Tm°C) [Arkell oligo number] | PCR program |
| --- | --- | --- | --- |
| Two-Primer Assays | | | |
| D6Mit268 | 5’- AGTCAGAATATGGCAAGTCAGTG -‘3 (60.9) | 5’- TTTCAGAGTCTTTCTTTCAGTATCTCC -‘3 (63.7) | TD65 |
| D6Mit235 | 5’- ATTTCTGTCGTGCCTGAGCT  -‘3 (58.4) | 5’- TCATGTGTGCTTGCTTGTGA -‘3 (56.4) | TD65 |
| D6Mit213 | 5’- TTGATCATAGTCAGATGAAGCACA -‘3 (60.3) | 5’- CTCAACACTGTAAAGCCTAAAATCA -‘3 (60.9) | TD65 |
| D6Mit201 | 5’- TTAGAGGAAGAGAACTGATAGAATGTG -‘3 (63.7) | 5’- ATTAACTTCAAGGAGAAGCCCC -‘3 (60.1) | TD65 |
| D6Mit83 | 5’- TTCTGTAAATTGCTAATCTGTCCA -‘3 (58.3) | 5’- TTGTATGCATTTAACAACTCAGGA -‘3 (58.3) | TD65 |
| D6Mit104 | 5’- CTCCAAATGCATGTGGACAC -‘3 (58.4) | 5’- CATCCCTCATGCCTCTGC -‘3 (58.4) | TD65 |
| D7Mit76 | 5’-  CATGAGCACGTGGAGAAAGA -‘3 (58.4) | 5’- CGTGGAAACCTGATAAACTGA -‘3 (57.5) | TD65 |
| D7Mit247 | 5’-  TCTTTTGACTTGATTTTGGCG -‘3 (55.4) | 5’- TGGAGGAGACATATCTTTGCG -‘3 (59.5) | TD65 |
| D7Mit276 | 5’-  CTGGGAGGAATGTTCTCCAA -‘3 (58.4) | 5’- AATGCCCAGTGTAGAAGAAACC -‘3 (60.1) | TD65 |
| D7Mit69 | 5’-  CCCACCAGAGATCACCAAGT -‘3 (60.5) | 5’- CACAATGAAGGCTGAAAGCA -‘3 (56.4) | TD65 |
| D8Mit155 | 5’-  TTGGACAGGGAAAATTCTGC -‘3 (56.4) | 5’- TGAGGACTTGCTTTAAGAGTACTCC -‘3 (64.1) | TD65 |
| D9Mit89 | 5’- CACATACAAGGATATACATACACAGGC -‘3 (65.1) | 5’- TCACAGGAGGTGGCAGAAAT -‘3 (58.4) | TD65 |
| D9Mit12 | 5’- ATTCAAGGGGCAGTACACAT -‘3 (56.4) | 5’- TGGTCCTGGTAAAACTGCCT -‘3 (58.4) | TD65 |
| D9Mit214 | 5’- AGCACAGGAAAAGGACGCTA -‘3 (58.4) | 5’- AACCTGTCTCTGTAAAACTATCTCCA -‘3 (62.9) | TD65 |
| FIC | 5’- cggaaggaggacatcag -‘3 (54.9) [1067] | 5’- GCTCAGCTTGGGTGACT -‘3 (54.9)[1068] | TD65 |
| FIT | 5’- CTGAAGAATTCCGGAGTCC -‘3 (57.5)[1053] | 5’- AGCACCAGCATGTTCTTGG -‘3 (57.5)[1054] | TD65 |
| Ube1 sex assay | 5’- GAGGTCATGAAGGTCAG -‘3 (52.4)[1002] | 5'- GGGCATAAACTTTCCAG -'3 (49.9)[1003] | TD60 |
| Three-Primer Assays | | | |
| FIN | 5’- CTGAAGAATTCCGGAGTCC -‘3 (57.5)[1053] | 5’- AGCACCAGCATGTTCTTGG -‘3 (57.5)[1054]  5’- ATCTCGTCGTGACCCATGG -‘3 (59.5)[1116] | TD65 |
| RRF | 5’- TCGTGATCTGCAACTCC -‘3 (52.4)[1124] | 5’- GGAAAATACTCCGAGGC -‘3 (52.4)[1126]  5’- GATGTGGAATGTGTGCG -‘3 (52.4)[1112] | TD65 |
| RRFU | 5’- TCGTGATCTGCAACTCC -‘3 (52.4)[1124] | 5’- GGAAAATACTCCGAGGC -‘3 (52.4)[ 1126]  5’- TCGTGCCGAATTCTTGG -‘3 (52.4)[ 1115] | TD65 |
| NLZ | 5’- ATGTGGACGTGACCGGACAGAACT -‘3 (66.9)[922] | 5’- CTGGATGTAGGCATGGTTGGTAGGAT -‘3 (67.9)[ 923]  5’- GGCCCTCACATTGCCAAAAGA -‘3 (61.2)[ 946] | TD65 |
| ND6 | 5’- TGAGCATCAAGAATCGTCCA -‘3 (56.4)[922] | 5’- AGGGCTCAAAGATCCGAAAT -‘3 (56.4)[ 997]  5’- TCACAGCCCAGACGCTG -‘3 (57.3)[ 1031] | TD65 |
| Z5N | 5’- TAAACGGCCAGATGCGC -‘3 (54.9)[1249] | 5’- AGGGCTCAAAGATCCGAAAT -‘3 (56.4)[ 1251]  5’- CTTGCATTCCTTTGGCG -‘3 (52.4)[ 277] | TD65 |
| Four-Primer Assays | | | |
| Mnet | 5’- CACATCAGACTAATCACCTCG -‘3 (59.5)[ 1059]  5’- CACACACACATTTCTCTGTCCA -‘3 (60.1)[748] | 5’- GCTGCAAGGCGATTAAGTTG-‘3 (58.4)[1057]  5’- TAGCTCAGTGCTTGCAAGGTTA -‘3 (60.1)[749] | TD65 |
| K5C | 5’- CGGTCGATGCAACGAGTGAT -‘3 (60.5)[ 1083]  5’- CACACACACATTTCTCTGTCCA -‘3 (60.1) [748] | 5’- CCACCGTCAGTACGTGAGAT -‘3 (60.5)[1084]  5’- TAGCTCAGTGCTTGCAAGGTTA -‘3 (60.1) [749] | TD65 |
| K5S | 5’- TACAGCTCCTGGGCAACGTG -‘3 (62.5)[ 1072]  5’- CACACACACATTTCTCTGTCCA -‘3 (60.1) [748] | 5’- CACAGCATTGGAGTCAGAAG -‘3 (58.4)[1073]  5’- TAGCTCAGTGCTTGCAAGGTTA -‘3 (60.1) [749] | TD65 |

Table S1: Primers and PCR conditions. Primer sequences for microsatellite markers obtained from the Mouse Genome Informatics website.
